# Role of protein tyrosine phosphatase receptor type M in epithelial ovarian cancer progression

**DOI:** 10.1186/s13048-023-01220-3

**Published:** 2023-07-04

**Authors:** Xiao Li, Wei Ding, Yang Rao, Pengpeng Qu

**Affiliations:** Department of Gynecological Oncology, Tianjin Central Hospital of Obstetrics and Gynecology, Tianjin Key Laboratory of Human Development and Reproductive Regulation, 156 Nankai Third Road, Nankai, Tianjin, 300100 P. R. China

**Keywords:** Carcinoma, Ovarian epithelial, Receptor-like protein tyrosine phosphatases, Gene expression profiling, Prognosis, Survival analysis

## Abstract

**Background:**

Epithelial ovarian cancer (EOC) is often diagnosed at advanced stages with low survival rates. Protein tyrosine phosphatase receptor type M (PTPRM) is involved in cancer development and progression; however, its role in EOC remains unclear. In this study,we aimed to detect PTPRM expression in ovarian epithelial tumors, analyze its relationship with the clinicopathological features and survival prognosis of patients with EOC, and provide a theoretical basis for new targets for EOC treatment. Fifty-seven patients with EOC treated at our hospital between January 2012–January 2014 were included; along with 18 borderline and 30 benign epithelial ovarian tumors and 15 normal ovarian and uterine tube tissue samples from patients surgically treated at our hospital during the same period. PTPRM expression was immunohistochemically detected, and we analyzed its relationship with clinicopathological features and prognosis. Associations between PTPRM expression and survival prognosis of patients with EOC were analyzed using the Gene Expression Profiling Interactive Analysis (GEPIA) and Kaplan–Meier Plotter databases.

**Results:**

PTPRM had the highest expression rates in normal ovarian and uterine tube tissues, followed by benign and borderline epithelial ovarian tumors; the lowest positive expression rate was observed in EOC tumors. PTPRM expression differed significantly among groups (*P* < 0.05). The positive PTPRM expression rate significantly decreased with age, progressing clinical stage, and tumor recurrence, and the larger the mass diameter, the higher the positive PTPRM expression rate. PTPRM expression was significantly lower in ovarian cancer compared with that in normal tissues in the GEPIA database (*P* < 0.05). The overall survival (OS) and disease-free survival(DFS) rates were higher in the PTPRM high-expression group, with statistically significant (*P* < 0.05) and insignificant (*P* > 0.05) differences, respectively. The OS rate of the high-expression group compared with the low-expression group in the Kaplan–Meier Plotter database was higher, although without statistical significance (*P* > 0.05), and progression-free survival(PFS) was higher with statistical significance (*P* < 0.05).

**Conclusion:**

PTPRM expression was low in patients with EOC, and the PTPRM positive-expression rate significantly decreased with progressing stages of EOC and tumor recurrence, suggesting that PTPRM acts as a tumor suppressor in EOC progression. Negative PTPRM expression may predict poor clinical outcomes in patients with EOC.

## Background

Ovarian cancer is the eighth most common cancer in women and has the highest mortality rate among gynecologic malignancies,accounting for nearly 50% of new cases reported annually by the American Cancer Society [1]. According to different histogenetic sources, ovarian cancer can be divided into four types—epithelial ovarian cancer (EOC), malignant germ cell tumors, ovarian sex gonad stromal tumors, and mixed tumors—among which EOC accounts for more than 90% of ovarian malignancies [2]. Because the ovaries are located deep within the pelvis and early lesions are difficult to detect, most ovarian cancer patients are diagnosed at an advanced stage when the tumor has already progressed.The standard treatment is cytoreductive surgery followed by platinum-based adjuvant chemotherapy. Treatment strategies for ovarian cancer have made great progress in recent years, including targeted therapies and immunotherapy. However, because of treatment resistance and the gap between preclinical findings and actual clinical outcomes, ovarian cancer poses a serious threat to women’s health. Therefore, to improve survival rates of ovarian cancer patients, exploring an effective therapeutic target is critical.

Protein tyrosine phosphatase (PTP) is involved in a variety of cellular functions and plays an important role in various physiological and pathological processes. Defects in tyrosine phosphorylation-mediated signaling events are associated with a variety of diseases such as cancer, autoimmune diseases, and diabetes/obesity. PTP appears as a specific regulatory factor for tyrosine phosphorylation in cancer cells [3]. PTP is a potential target for cancer therapy. Protein tyrosine phosphatase receptor type M (PTPRM) is a receptor-type tyrosine phosphatase involved in the development and progression of several malignancies; however, the role of PTPRM in EOC remains unclear. In this study, we aimed to detect the expression of PTPRM in ovarian epithelial tumors, analyze its relationship with the clinicopathological features and survival prognosis of patients with EOC, and provide a theoretical basis for new targets for EOC treatment.

## Results

### Expression of PTPRM in different ovarian epithelial tumors and normal ovarian and uterine tube tissues

**Fig. 1:**
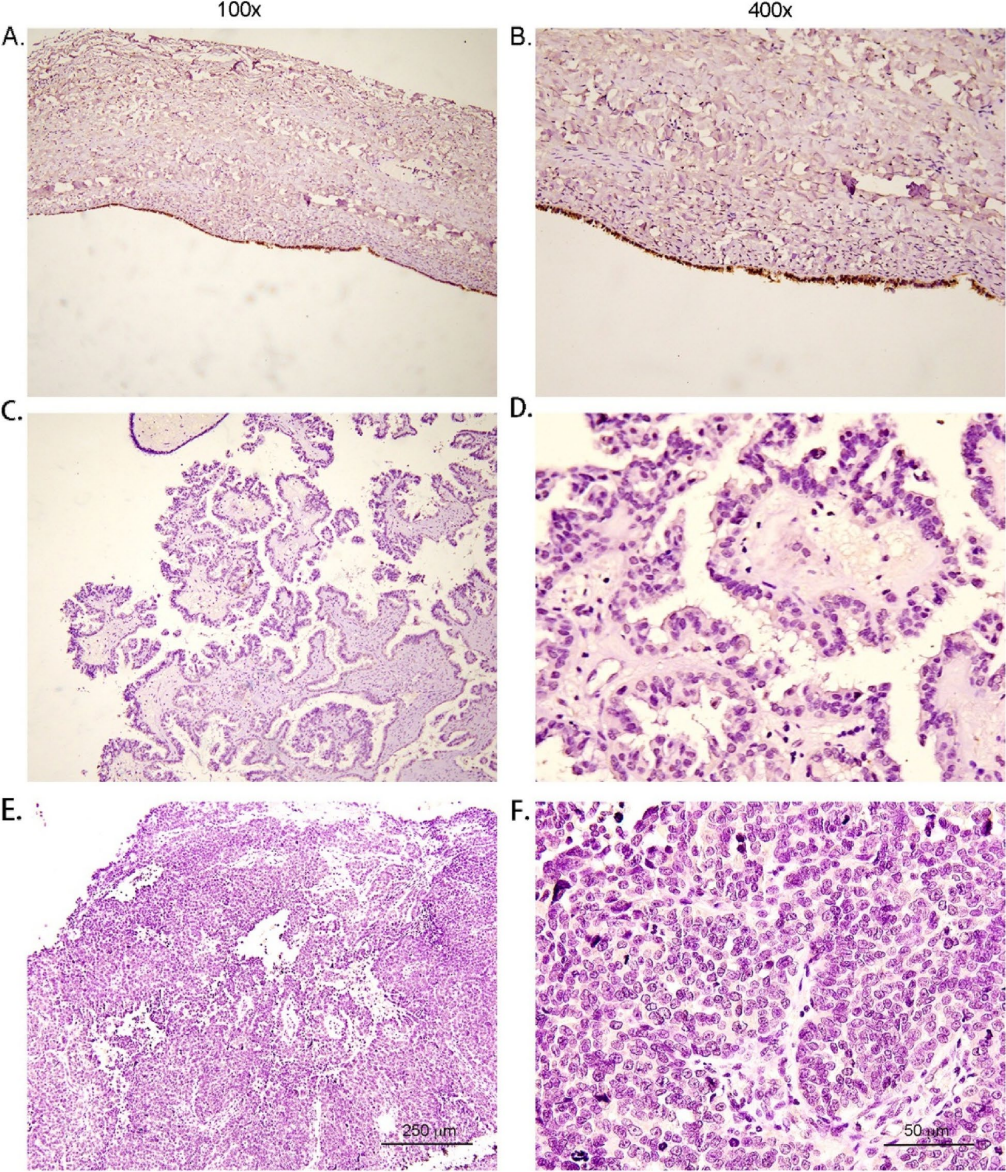
The expression of PTPRM in benign ovarian epithelial tumors (**A**, **B**), borderline epithelial ovarian tumors (**C**, **D**) and EOC (**E**, **F**),as detected by immunohistochemistry. The positive expression rate of PTPRM in benign and borderline epithelial ovarian tumors was higher than that in EOC. Abbreviations: EOC, epithelial ovarian cancer; PTPRM, protein tyrosine phosphatase receptor type M

**Fig. 2:**
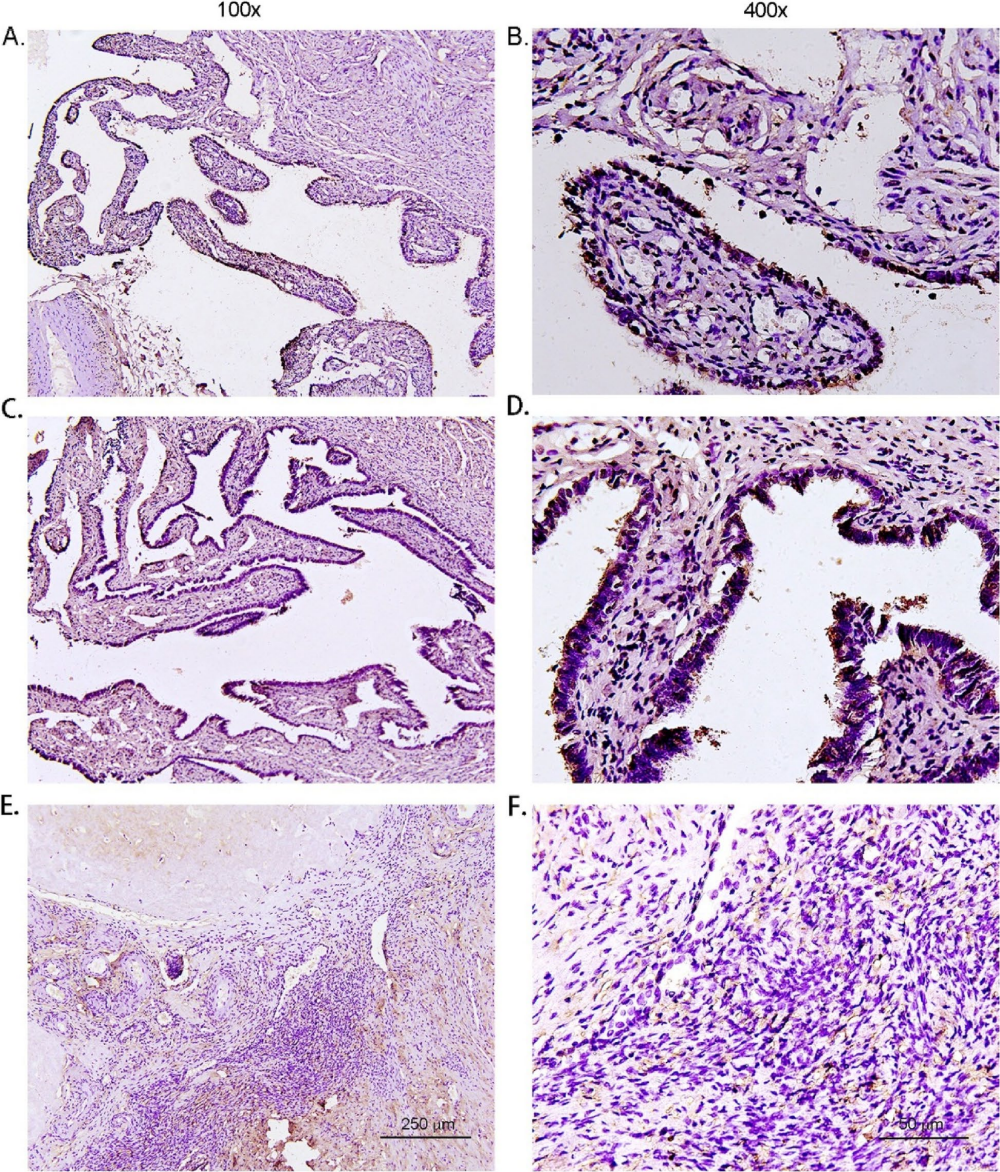
Expression of PTPRM in normal ovarian and uterine tube tissues detected by immunohistochemistry. PTPRM had the highest positive expression rate in normal uterine tube epithelial tissues (**A**, **B**, **C**, **D**) and normal ovarian tissues (**E**, **F**). Abbreviations:PTPRM, protein tyrosine phosphatase receptor type M

Tissue samples from several cases of ovarian epithelial tumors—including 57 cases of EOC, 18 of borderline epithelial ovarian tumors, 30 of benign ovarian epithelial tumors, and 15 cases of normal ovarian and uterine tube tissues—were stained by immunohistochemistry, and the results showed that PTPRM had the highest positive expression rate in normal ovarian and uterine tube tissues, followed by benign ovarian epithelial tumors and borderline epithelial ovarian tumors;the lowest positive expression rate was observed in EOC. The expression of PTPRM differed significantly among the four groups (*P* < 0.05) (Table 1, Figs. 1 and 2).

**Table 1:** Expression of PTPRM in ovarian epithelial tumors of different natures and normal ovarian and uterine tube tissues

	Negative		Positive		Total	*P-value*
	Number of cases (n)	Percentage (%)	Number of cases (n)	Percentage (%)		
EOC	48	84.2	9	15.8	57	0.000
Borderline epithelial ovarian tumor	9	50.0	9	50.0	18	
Benign ovarian epithelial tumor	6	20.0	24	80.0	30	
Normal ovarian and uterine tube tissue	0	0.0	15	100.0	15	
Total	63		57		120	

*EOC* epithelial ovarian cancer, *PTPRM* protein tyrosine phosphatase receptor type M

### Relationship between PTPRM expression and clinicopathological parameters of EOC

To further investigate the role of PTPRM in the progression of EOC, the relationship between the expression of PTPRM and clinicopathological parameters—such as patient's age, clinical stage, pathological type, age of menarche, age of menostasis, number of pregnancies, family history of malignancy, presence of medical comorbidities, serum tumor marker levels (cancer antigen [CA]125, CA19-9, human epididymis protein 4 [HE4]), presence of ascites, tumor diameter, bilateral mass or not, lymph node metastasis, and disease recurrence—were analyzed separately. The χ^2^ test was used for a two-by-two comparison between the groups, and *P* < 0.05 was considered statistically significant.

**Fig. 3:**
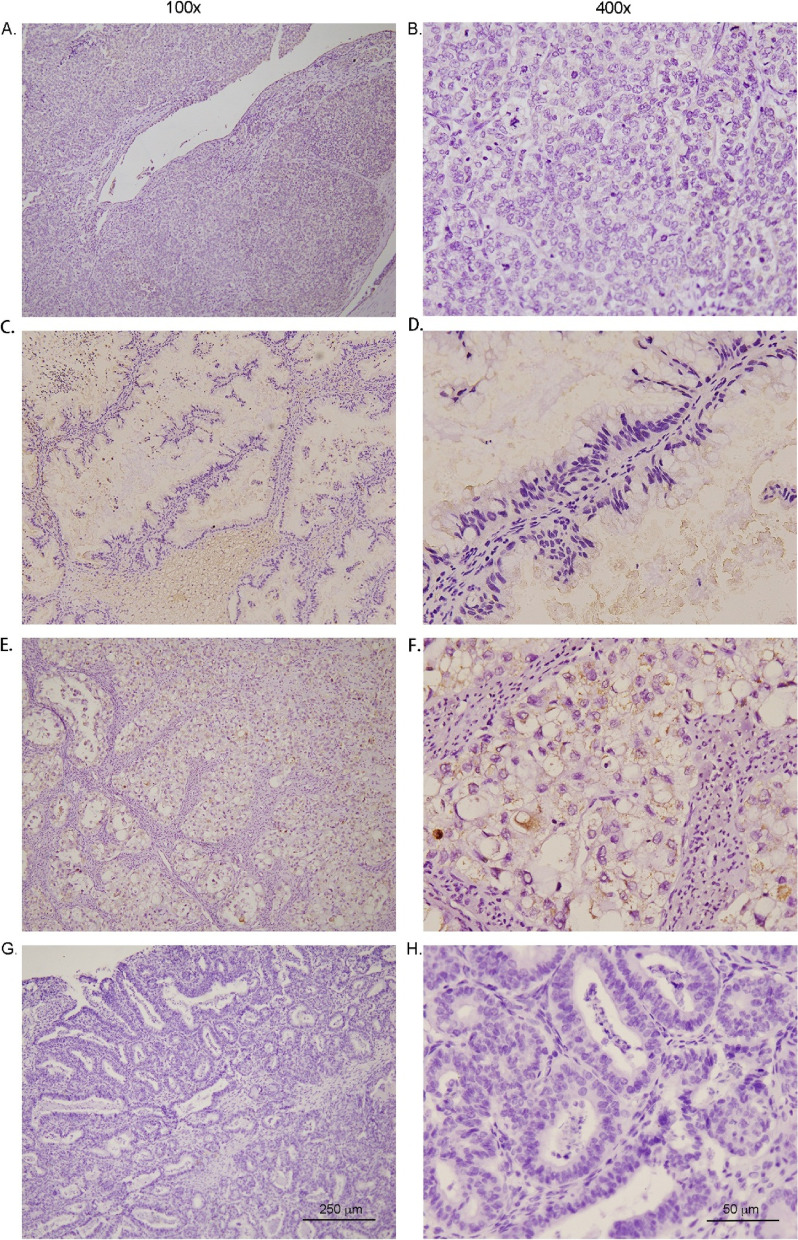
Immunohistochemical detection of PTPRM expression in different pathological types of epithelial ovarian carcinoma—(**A**, **B**) Serous carcinoma; (**C**, **D**) mucinous carcinoma; (**E**, **F**) clear cell carcinoma; and (**G**, **H**) endometrioid carcinoma. The expression difference of PTPRM is not significant among various pathological types. Abbreviations: PTPRM, protein tyrosine phosphatase receptor type M

The results of this study showed that the expression of PTPRM in EOC was significantly different in patients varying in terms of age, clinical stage, the maximum diameter of the mass, and tumor recurrence in the subgroup; however,the differences were not significant in patients varying in the factors of pathological type, age of menarche, age of menostasis,number of pregnancies, family history, presence of medical comorbidities, tumor markers, presence of ascites, unilateral or bilateral masses, and presence of lymph node metastasis (Table 2, Fig. 3).

**Table 2:** Relationship between the expression of PTPRM and clinicopathological parameters of EOC

Clinicopathological parameters	Total number of cases	Negative		Positive		*P-value*
		Number of cases (*n* = 48)	Percentage (%)	Number of cases (*n* = 9)	Percentage (%)	
Age (years)						0.043
< 50	21	15	71.4	6	28.6	
≥ 50	36	33	91.7	3	8.3	
Clinical stages						0.031
I + II	32	24	75.0	8	25.0	
III + IV	25	24	96.0	1	4.0	
Pathological type						0.339
Serous carcinoma	32	29	90.6	3	9.4	
Mucinous carcinoma	7	6	85.7	1	14.3	
Clear cell carcinoma	9	6	66.7	3	33.3	
Endometrioid carcinoma	9	7	77.8	2	22.2	
Age of menarche (years)						0.227
< 15	34	27	79.4	7	20.6	
≥ 15	23	21	91.3	2	8.7	
Age of menostasis (years)						0.863
< 50	17	15	88.2	2	11.8	
≥ 50	20	18	90.0	2	10.0	
Number of pregnancies (times)						0.837
< 1	3	2	66.7	1	33.3	
≥ 1	54	46	85.2	8	14.8	
Family history of malignant tumors						0.110
None	46	37	80.4	9	19.6	
Yes	11	11	100.0	0	0	
Medical comorbidities						0.802
None	40	34	85.0	6	15.0	
Yes	17	14	82.4	3	17.6	
CA125 (U/ml)						0.587
< 500	40	33	82.5	7	17.5	
≥ 500	17	15	88.2	2	11.8	
CA19-9 (U/ml)						0.059
< 27	35	32	91.4	3	8.6	
≥ 27	22	16	72.7	6	27.3	
HE4 (pmol/L)						0.251
< 140	29	26	89.7	3	10.3	
≥ 140	28	22	78.6	6	21.4	
Ascites						0.702
None	16	13	81.3	3	18.7	
Yes	41	35	85.4	6	14.6	
Tumor diameter (cm)						0.040
< 10	24	23	95.8	1	4.2	
≥ 10	33	25	75.8	8	24.2	
Whether bilateral						0.051
Yes	15	15	100.0	0	0	
No	42	33	78.6	9	21.4	
Pathological lymph node metastasis (pN)						0.157
None	48	39	81.3	9	18.7	
Yes	9	9	100.0	0	0	
Whether recur						0.026
Yes	18	18	100.0	0	0	
No	39	30	76.9	9	23.1	

*CA* cancer antigen, *EOC* epithelial ovarian cancer, *HE4* human epididymis protein 4, *PTPRM* protein tyrosine phosphatase receptor type M

### PTPRM expression and survival of patients with EOC

**Fig. 4:**
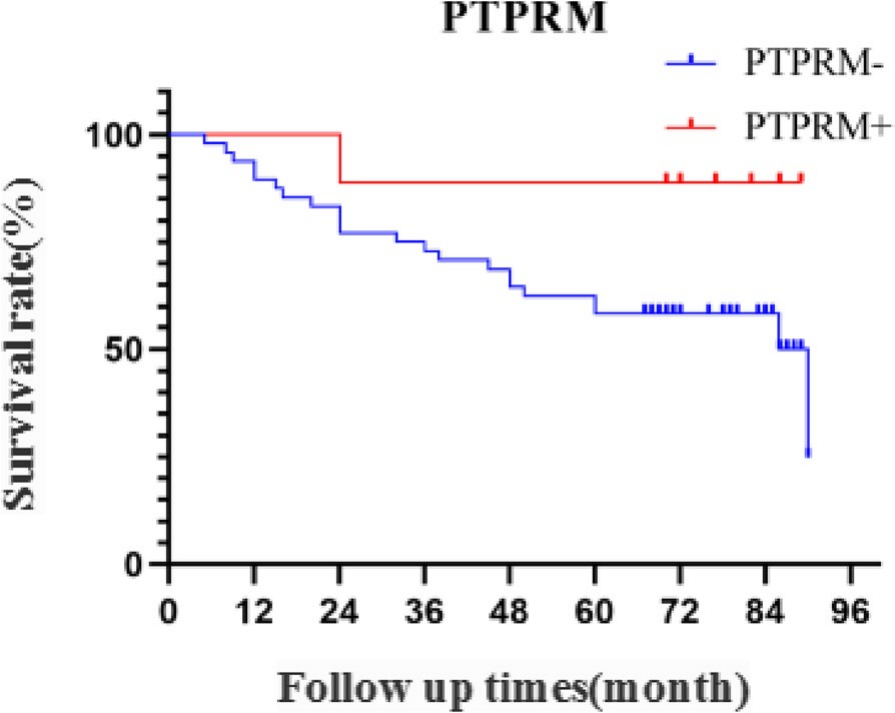
Survival curves of patients with PTPRM expression.Patients with positive PTPRM expression had a higher survival rate than patients with negative PTPRM expression. Abbreviations: PTPRM, protein tyrosine phosphatase receptor type M

Fifty-seven patients with EOC were followed up for > 60 months, with no loss of follow-up; 23 deaths were recorded during the follow-up period. The mean survival time was 67.263 ± 4.200 months. During the follow-up period, there was one death and eight survivors in the PTPRM expression-positive group and 22 deaths and 26 survivors in the PTPRM expression-negative group (Table 3). P25 survival time for patients in the expression-negative group was 34.000 months (95% confidence interval: 15.000, 50.000), with a mean survival time of 64.625 months (standard error: 4.691).Survival time for patients who died in the expression-positive group was 24 months; eight patients did not die after a mean follow-up of 78.750 ± 6.541 months.We observed that patients with positive PTPRM expression had a higher survival rate than patients with negative PTPRM expression.The survival curves of patients with different PTPRM expressions are shown in Fig. 4, although no statistical difference was observed between the two groups (Log-rank = 2.878, *P* = 0.090).

**Table 3:** Expression of PTPRM and survival status

Diagnosis	PTPRM Expression	Total number of cases	Number of surviving cases (%)	Survival rate (%)			
				1 year	2 years	3 years	5 years and above
EOC	Positive	9	8 (88.89)	100.00	88.89	88.89	88.89
	Negative	48	26 (54.17)	89.58	77.08	72.92	58.33

*EOC* epithelial ovarian cancer, *PTPRM* protein tyrosine phosphatase receptor type M

### Analysis of the effects of PTPRM expression and various clinicopathological parameters on the prognosis of patients with EOC

The results of univariate Cox analysis showed the effects of patient age, clinical stage, presence of ascites, CA125, HE4, and whether lymphatic metastasis had statistically significant effects on patient survival in this study (*P* < 0.05). The effect of PTPRM expression on prognosis was not statistically significant (*P* = 0.128) (Table 4).

**Table 4:** Univariate Cox regression analysis

Factors	*β*	*SE*	*HR (95% CI)*	***P-value***
PTPRM	-1.559	1.024	0.210(0.028,1.565)	0.128
Age	1.113	0.557	3.042(1.020,9.072)	**0.046**
CA125	1.517	0.436	4.559(1.94,10.716)	**0.001**
Clinical stages	2.567	0.626	13.022(3.821,44.379)	**< .0001**
Ascites	1.232	0.621	3.428(1.015,11.571)	**0.047**
Tumor diameter	-0.734	0.434	0.480(0.205,1.124)	0.091
Family history	0.561	0.484	1.752(0.679,4.521)	0.246
Age of menostasis	-0.228	0.461	0.796(0.323,1.964)	0.620
Age of menarche	0.475	0.427	1.607(0.697,3.709)	0.266
Comorbidities	0.288	0.441	1.334(0.563,3.164)	0.513
HE4	1.529	0.514	4.613(1.686,12.622)	**0.003**
CA19-9	-0.932	0.510	0.394(0.145,1.069)	0.068
Lymphatic metastasis	1.638	0.456	5.144(2.106,12.566)	**< 0.001**

*CA* cancer antigen, *CI* confidence interval, *HE4* human epididymis protein 4, *HR* hazard ratio, *PTPRM* protein tyrosine phosphatase receptor type M, *SE* standard error

Variables that were statistically significant in the univariate analysis were included in the Cox multiple regression model, and the step-by-step method was used to screen the independent variables (α = 0.05). The results showed a statistically significant effect of clinical stage and HE4 on patient prognosis (Table 5).

**Table 5:** Multivariable analysis of prognosis of patients with EOC

Factors	*β*	*SE*	*Wald χ*^*2*^	*HR (CI 95%)*	***P-value***
Clinical Stages	2.547	0.636	16.017	12.768 (3.668,44.443)	**< 0.001**
HE4	1.456	0.530	7.545	4.290 (1.518,12.129)	**0.006**

*CI* confidence interval, *EOC* epithelial ovarian cancer, *HE4* human epididymis protein 4, *HR* hazard ratio, *SE* standard error

Compared with early-stage cancer, patients with advanced cancer had a worse prognosis, with a 12.768 times(3.668, 44.443) higher risk of death than early-stage cancer and 4.290 times(1.518, 12.129) higher risk of death in patients with abnormal HE4 than in normal patients.

### Application of database to analyze the expression of PTPRM in ovarian malignant tumors and its relationship with survival prognosis

**Fig. 5:**
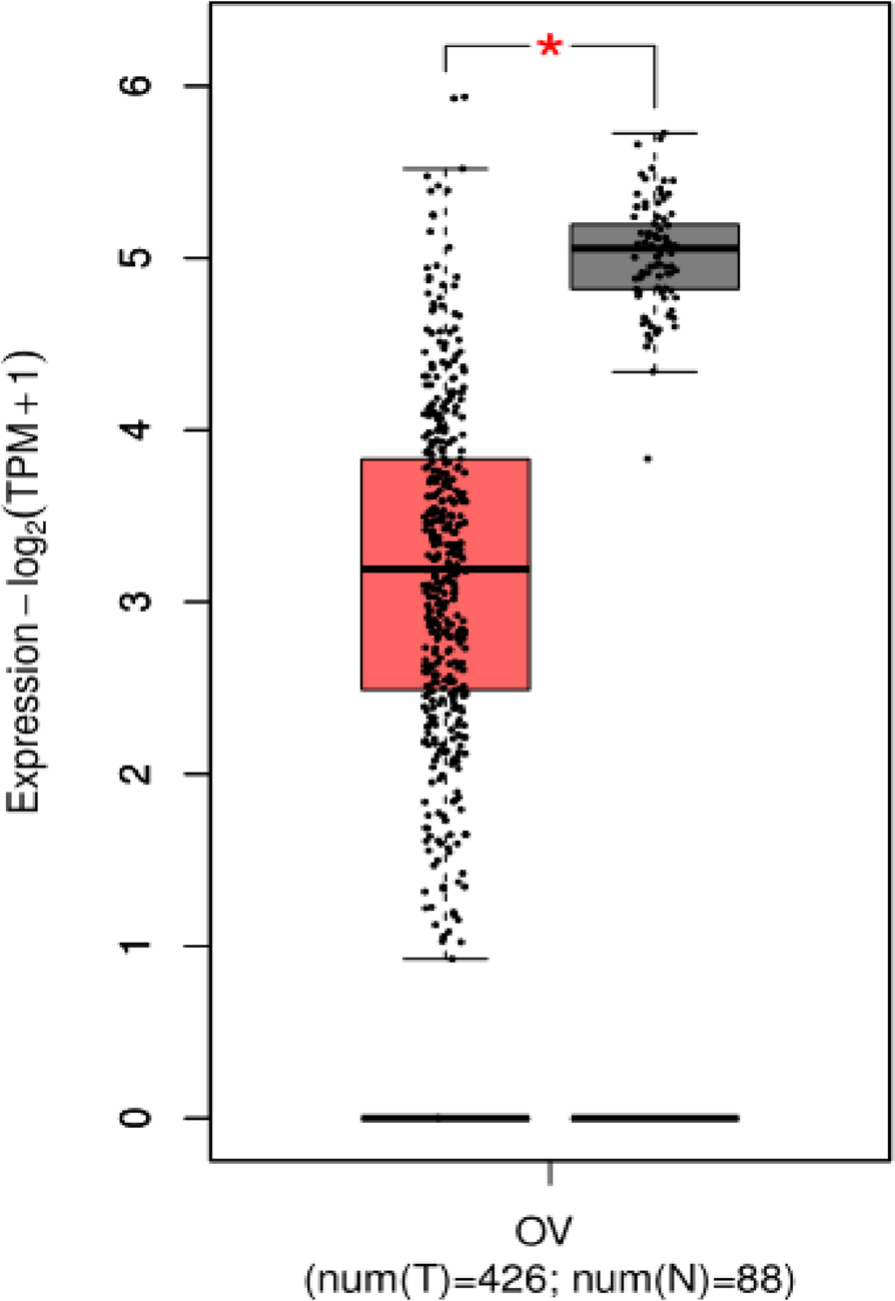
Expression of PTPRM in ovarian cancer in the GEPIA database. The expression of PTPRM was significantly lower in ovarian cancer tissues than that in normal ovarian tissues. Red represents the tumor samples and gray represents the normal samples. Abbreviations: GEPIA, Gene Expression Profiling Interactive Analysis; num(N), number(Normal); num(T), number(Tumor); OV, ovary; PTPRM, protein tyrosine phosphatase receptor type M; TPM, Transcripts Per Million; *,P < 0.05

The expression of PTPRM in ovarian cancer and normal ovarian tissues was analyzed using the online database GEPIA (http://www.sci666.net/29414.html), and it was found that the expression of PTPRM was significantly lower in ovarian cancer tissues than that in normal ovarian tissues (*P* < 0.05) (Fig. 5).

**Fig. 6:**
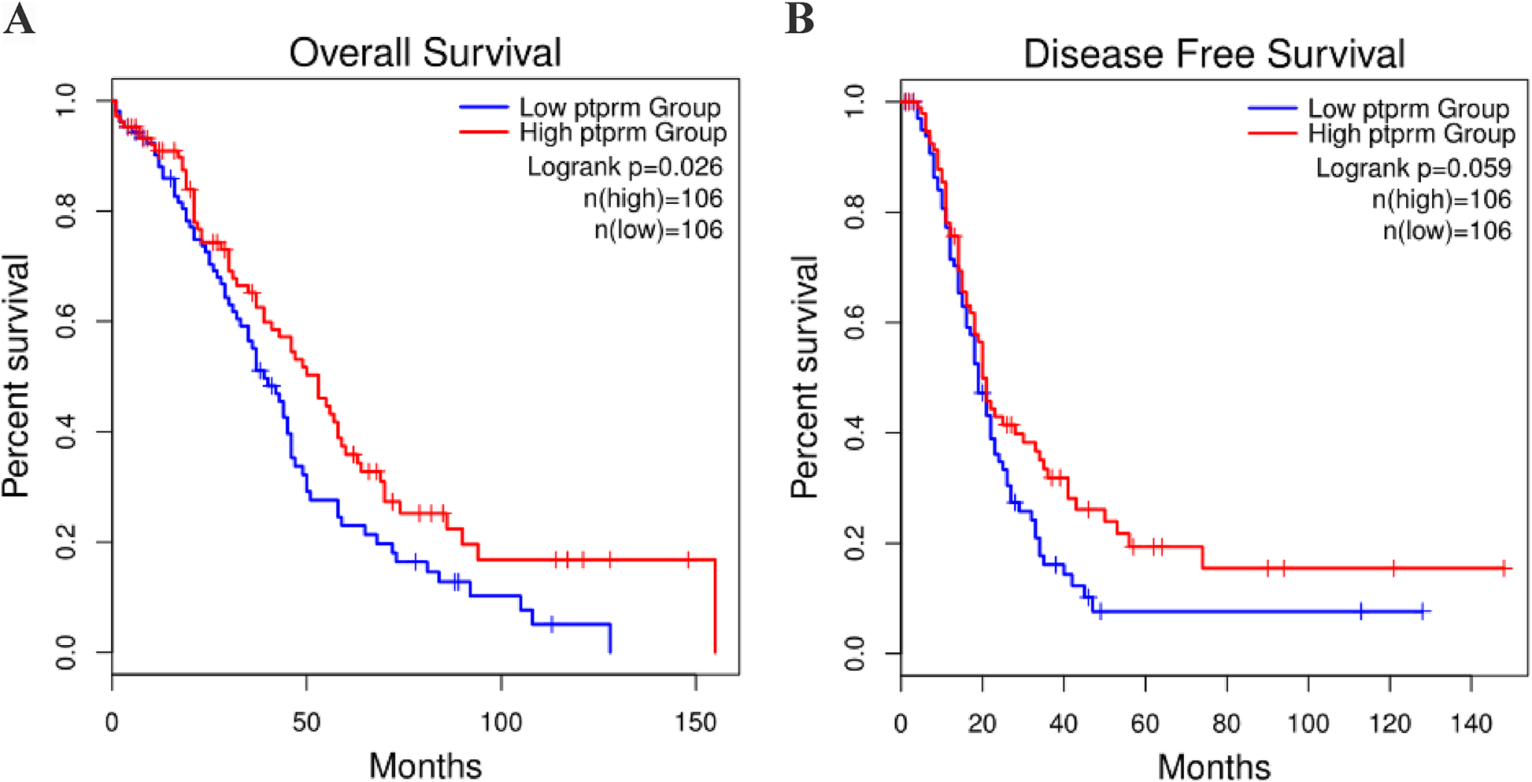
The GEPIA database showed that OS (**A**) and DFS (**B**) of the PTPRM high-expression group were higher than that of the PTPRM low-expression group. The red line indicates patients with PTPRM high-expression and the blue line indicates patients with PTPRM low-expression. Abbreviations: GEPIA, Gene Expression Profiling Interactive Analysis; PTPRM, protein tyrosine phosphatase receptor type M; OS, overall survival; DFS, disease-free survival

**Fig. 7:**
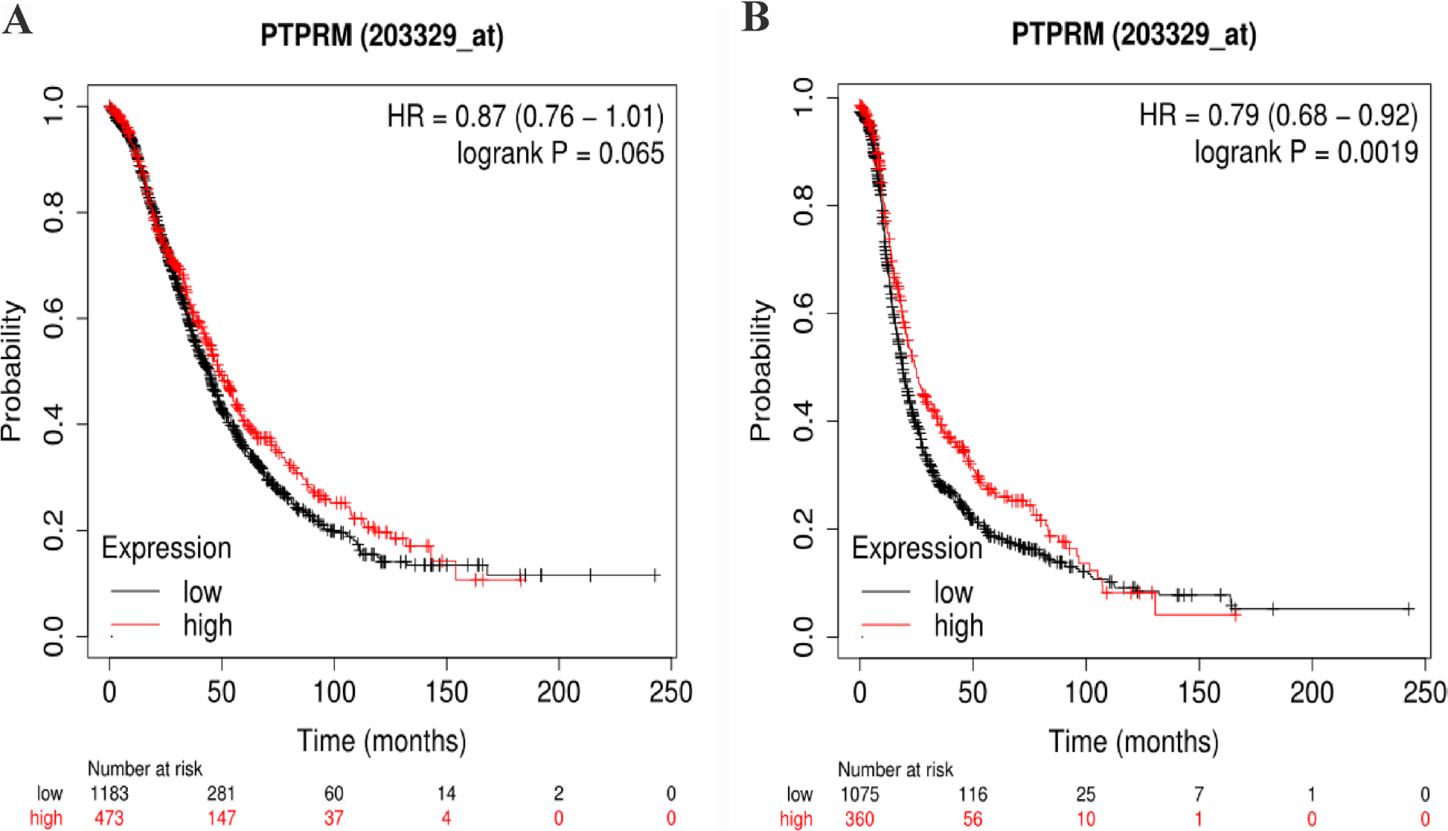
Relationship between PTPRM expression and survival prognosis of ovarian cancer in Kaplan–Meier Plotter database. OS (**A**) and PFS (**B**) were higher in the PTPRM high-expression group than in the PTPRM low-expression group. The red line indicates patients with PTPRM high-expression and the black line indicates patients with PTPRM low-expression. Abbreviations: HR, hazard ratio; PTPRM, protein tyrosine phosphatase receptor type M; OS, overall survival; PFS, progression-free survival

The relationship between the expression of PTPRM and the survival prognosis of ovarian cancer patients was analyzed using the GEPIA database, and it was found that the overall survival(OS) of the PTPRM high-expression group was higher than that of the PTPRM low-expression group, and the difference was statistically significant (*P* < 0.05). Moreover,disease-free survival(DFS) rate of the PTPRM high-expression group was higher than that of the PTPRM low-expression group, but the difference was not statistically significant (*P* > 0.05) (Fig. 6). The prognostic impact of PTPRM on the survival of patients with ovarian cancer was analyzed using the Kaplan–Meier Plotter database (http://kmplot.com/analysis/), and it was found that the OS of the PTPRM high-expression group was higher than that of the low-expression group, but the difference was not statistically significant (*P* > 0.05), as shown in Fig. 7A. Progression-free survival(PFS) rate was higher in the PTPRM high-expression group than in the PTPRM low-expression group, and the difference was statistically significant (*P* < 0.05), as shown in Fig. 7B.

## Discussion

PTPRM is a transmembrane receptor-like PTP(RPTP) that belongs to the R2B subfamily of RPTPs [4]. PTPRM gene is located on chromosome 18p11.2. PTPRM has both cell adhesion and signaling capabilities. PTPRM has a large extracellular region that includes a MAM (Meprin, A5, PTP mu) domain, an immunoglobulin (Ig) domain, and four fibronectin type III(FNIII) repeat sequences [5–7]. The intracellular region contains a near membrane domain, followed by a catalytic active tyrosine phosphatase domain and a second inactive domain. The intracellular membrane domain contains a region homologous to the conserved intracellular domain of cadherin, and only the membrane proximal PTP domain is catalytically active. PTPRM binds to the FN1 and 2 domains of other molecules on adjacent cells through the MAM and Ig domains in the extracellular region, promoting cell–cell interactions in the same way, which is independent of phosphatase activity in the intracellular region [8, 9]. By its phosphatase activity, signals from outside the cell are transduced into the intracellular environment.It is precisely because of the above structural features that PTPRM is essential for in-cell growth, activation, and signal transduction [10, 11].

PTPRM is expressed in neurons, glial cells, epithelial cells, and the prostate [12–14]. It is extensively involved in the development and progression of certain malignancies. PTPRM has an important role in tumorigenesis as a tumor suppressor gene. During tumor development, PTPRM affects cell proliferation, survival, apoptosis, vesicular transport, adhesion, migration, and invasion.

Barazeghi et al. [15] showed that PTPRM is undetectable or expressed at very low levels in small intestinal neuroendocrine tumors (SI-NET). Sudhir et al. [16] confirmed the absence and downregulation of PTPRM expression in colonic adenomas and carcinomas. Burgoyne et al. [13] showed a significant reduction in PTPRM protein expression in glioblastoma multiforme. A certain level of PTPRM expression was maintained in low-grade astrocytoma samples compared with glioblastoma multiforme. Studies addressing the role of PTPRM in human glioblastoma multiforme have also shown that PTPRM expression is frequently downregulated in this malignancy [11, 13]. These results suggest that loss of PTPRM protein expression may be an important event in glioma progression. Sun et al. [17] explored the expression of PTPRM in breast cancer and showed that its transcripts were significantly reduced in hypodifferentiated and moderately differentiated tumors compared with well-differentiated tumors. Patients with lower PTPRM expression had shorter disease-free survival compared to those with higher PTPRM expression levels. Decreased expression of PTPRM in breast cancer was associated with poor prognosis and was negatively correlated with disease-free survival. However, Bae et al. [18] found that PTPRM expression was increased in gastric cancer, especially in poorly cohesive carcinoma and that is related to unfavorable prognosis.

One of the mechanisms by which cells lose contact inhibition of growth,and may promote tumorigenesis is the protein hydrolysis of cell–cell adhesion receptors, which alters the ability of cells to respond to normal extracellular signals. The proteolytic cleavage of PTPRM leads to the shedding of the extracellular domain, which results in cells losing contact with each other [19]. Studies in gliomas suggest that PTPRM cleavage promotes malignant glioma development in at least two ways: (1) the disassembly of its extracellular domain, which disrupts intercellular and cell–matrix adhesion, and (2) the reduction of the intrinsic phosphatase activity of the intracellular domain, which antagonises the RTK signaling pathway [20].

As mentioned earlier, PTPRM plays an important role in the development of a variety of malignancies. However, the expression characteristics of PTPRM in EOC and its clinical/prognostic significance are unclear. Therefore, we investigated the expression and clinicopathological significance of PTPRM in patients with EOC and its relationship with survival prognosis. In this study, the expression of PTPRM in different ovarian epithelial tumors and normal ovarian and uterine tube tissues was detected using immunohistochemistry, and the results showed that the highest positive expression rate of PTPRM was found in normal ovarian and uterine tube tissues, followed by benign ovarian epithelial tumors and borderline epithelial ovarian tumors;the lowest positive expression rate was found in EOC, with significant differences(*P* < 0.05), suggesting that PTPRM may be an important molecular influencing factor in the progression of EOC. In addition, this study also analyzed the expression of PTPRM in ovarian cancer and normal ovarian tissues through an online database and found that the expression of PTPRM in ovarian cancer tissues was significantly lower than that in normal ovarian tissues, further supporting the role of PTPRM as a tumor suppressor.

To further investigate the role of PTPRM in the progression of EOC, the relationship between the expression of PTPRM and clinicopathological parameters—such as patient's age, clinical stage, pathological type, age of menarche, age of menostasis, number of pregnancies, family history of malignancy, presence of medical comorbidities, serum tumor marker levels (CA125, CA19-9, HE4), presence of ascites, tumor diameter,bilateral mass or not, lymph node metastasis, and disease recurrence—were analyzed separately. The results showed that the expression of PTPRM in EOC was significantly different in the comparison of patients' age, clinical stage, maximum diameter of the mass,and whether the tumor recurred in the subgroup, whereas the differences were not significant in the comparison of the factors of pathological type, age of menarche, age of menostasis, number of pregnancies, family history, presence of medical comorbidities, tumor markers, presence of ascites, unilateral and bilateral masses, and presence of lymph node metastasis. Among them, the positive expression rate of PTPRM decreased with progressing stages of EOC and tumor recurrence, and the difference was significant, indicating that PTPRM may play a role in the progression and recurrence of EOC. Nakanishi [21] showed that the tumor diameter of ovarian cancer was closely related to the stage of the disease. This study showed that PTPRM was associated with the diameter of the mass, and further analysis revealed that a majority of the patients with a mass diameter < 10 cm were in clinical stage III–IV, and a majority of the patients with mass diameter ≥ 10 cm were in clinical stage I–II, which explained the higher rate of positive PTPRM expression with larger masses.

Laczmanska et al. [22] showed the effects of deletion of the chromosome 18 region containing PTPRM, suggesting the practical value of assessing the status of PTP receptors as a prognostic factor in colon cancer. Sahni [23] showed reduced plasma PTPRM in patients with a poor prognosis of pancreatic ductal adenocarcinoma, suggesting that PTPRM could be used as a new blood-based biomarker to predict the prognosis of pancreatic ductal adenocarcinoma. In this study, we analyzed the relationship between PTPRM expression and the survival prognosis of ovarian cancer patients through an online database and found that the OS of the group with PTPRM high-expression was significantly higher than that of the group with PTPRM low-expression.Meanwhile, based on the analysis of the expression of PTPRM and survival status in 57 patients with EOC, we found that patients with positive expression of PTPRM had a higher survival rate compared with those with negative expression of PTPRM, although the difference was not statistically significant. Analysis of the effect of each clinicopathological feature on the prognosis of ovarian cancer revealed that there was a statistically significant effect of patient age, clinical stage, presence of ascites, CA125, HE4, and lymphatic metastasis on patient survival, while the effect of PTPRM expression on prognosis was not statistically significant, which may be due to the small sample size. Negative expression of PTPRM may predict poor clinical outcomes in patients with EOC.

## Conclusions

The expression of PTPRM was low in patients with EOC, and the positive rate of PTPRM expression significantly decreased with progressing stages of EOC and tumor recurrence, suggesting the role of PTPRM as a tumor suppressor in the progression of EOC. Negative expression of PTPRM may predict poor clinical outcomes in patients with EOC.

## Material and methods

### Clinical specimen collection

Fifty-seven patients with EOC, hospitalized and surgically treated in our hospital from January 2012 to January 2014,were selected for this study. In addition, 18 patients with borderline epithelial ovarian tumors, 30 patients with benign epithelial tumors, and 15 patients with normal ovarian and uterine tube tissues,all surgically treated in our hospital during the same time period,were selected as the control group. All patients had complete clinical information (including age, age of menarche, age of menostasis, menstrual history, marital history, family history, presence of medical comorbidities, tumor markers, date of surgery, mode of surgery, tumor diameter, presence/ absence of bilateral mass, presence of ascites, lymph node metastasis, clinical stage,histological type, follow-up recurrence, and date of death); sample specimens collected from all patients were diagnosed pathologically by our pathologists.

We included patients who: (1) underwent tumor cytoreductive surgery and the diagnosis of EOC was confirmed after surgery; (2) did not receive neoadjuvant therapy before surgery; (3) did not have EOC combined with other malignant tumors; and (4) underwent regular follow-up after surgery with a follow-up period > 60 months.

### Follow up

The follow-up deadline to record the patient's status (survival, recurrence, or death) at the last follow-up visit was September 2019. The presence of recurrence and metastasis was determined based on the patient's clinical manifestations, tumor marker test results, and imaging examinations.

### Immunohistochemistry

Five-micrometer sections of paraffin-embedded human EOCs, borderline tumors, benign tumors, and normal ovarian tissues were prepared for staining. After dewaxing, the sections were rehydrated, then antigen retrieval and endogenous peroxidase blocking were performed. The slides were incubated with monoclonal antibodies (PTPRM: Santa Cruz, 1:200) overnight at 4 °C. The sections were then incubated with biotinylated anti-mouse secondary antibody (1:100) followed by horseradish peroxidase-streptavidin. Antigens were detected with peroxidase substrate and counterstained with hematoxylin.The primary antibody was replaced with phosphate buffered saline(PBS) as the negative control.Immunohistochemical staining of all sections was performed under the same conditions and at the same staining time. PTPRM was localized in the cytoplasm and/or nucleus, and cells with brownish-yellow coloration in the cytoplasm and/or nucleus were considered positive cells.Each specimen was randomly selected from 10 fields of view under a 400 × light microscope, and the number of positive cells in the 100 cells of the fields was counted(If the total number of cells in one field was less than 100, then 100 cells were counted in the adjacent two fields). The average number of positive cells was taken as the positive percentage, and if the number was greater than 30%, it was considered positive.

### Statistical methods

Data were statistically analyzed using SPSS software (version 22.0;IBM Corp., Armonk, NY, USA). The differences in each clinical parameter between the positive and negative PTPRM expression groups were determined using the χ^2^ test and Fisher's exact test. Patient survival time was described using the mean and standard deviation. The Kaplan–Meier survival curve method was used to compare the survival curves of the PTPRM expression-positive group with those of the expression-negative group; the Cox regression model was used to verify the effect of PTPRM expression on the prognosis of patients with EOC. Bioinformatics statistical analysis was performed using the statistical software within the database. *P* < 0.05 indicated statistically significant differences.

## Data Availability

All data generated or analyzed during this study are included in this article.

## Abbreviations

CA: Cancer antigen
EOC: Epithelial ovarian cancer
GEPIA: Gene Expression Profiling Interactive Analysis
HE4: Human epididymis protein 4
OS: Overall survival
DFS: Disease-free survival
PFS: Progression-free survival
PTP: Protein tyrosine phosphatase
PTPRM: Protein tyrosine phosphatase receptor type M
FNIII: Fibronectin type III
